# Polyploidy and the Evolution of Complex Traits

**DOI:** 10.1155/2012/292068

**Published:** 2012-07-30

**Authors:** Lukasz Huminiecki, Gavin C. Conant

**Affiliations:** ^1^CMB, Karolinska Institute, 17177 Stockholm, Sweden; ^2^DBB, Stockholm University, 10691 Stockholm, Sweden; ^3^MU Informatics Institute, University of Missouri, Columbia, MO 65211, USA; ^4^Division of Animal Sciences, University of Missouri, Columbia, MO 65211-5300, USA

## Abstract

We explore how whole-genome duplications (WGDs) may have given rise to complex innovations in cellular networks, innovations that could not have evolved through sequential single-gene duplications. We focus on two classical WGD events, one in bakers' yeast and the other at the base of vertebrates (i.e., two rounds of whole-genome duplication: 2R-WGD). Two complex adaptations are discussed in detail: aerobic ethanol fermentation in yeast and the rewiring of the vertebrate developmental regulatory network through the 2R-WGD. These two examples, derived from diverged branches on the eukaryotic tree, boldly underline the evolutionary potential of WGD in facilitating major evolutionary transitions. We close by arguing that the evolutionary importance of WGD may require updating certain aspects of modern evolutionary theory, perhaps helping to synthesize a new evolutionary systems biology.

## 1. Introduction

Characteristic changes in karyotype number have allowed researchers to infer polyploidy events for many decades [1]. It was thus with a reasonably long history of research that Susumo Ohno was able to suggest that polyploidy was a vital route to evolutionary innovation [2]. Ohno was of course a forceful proponent of a general role for duplication in evolution: writing that “[if evolution occurred only through changes allele frequencies] … from a bacterium only numerous forms of bacteria would have emerged […B]ig leaps in evolution required the creation of new gene loci with previously nonexistent functions” [2]. What is less obvious on first reading is his distinction between the role played by WGD and that played by other, smaller scale, duplications (or SSDs). While the differences in the scales of these events are self-evident, there are at least two other features of WGD that are critical in giving rise to these differing roles. The first is that, as many authors have reported, particular functional classes of genes (e.g., transcription factors, kinases, ribosomal proteins, and cyclins) are duplicated by WGD more frequently than by SSD [3–8]. Ohno had in fact explored the most likely reason for this difference: “hub” genes with many interactions with other loci, be those interactions regulatory, protein interaction or metabolic, will tend to respond poorly to a change in copy number. As a result, they will tend to survive in duplicate after WGD but will not survive after smaller scale events [2, 5, 9–11]. This idea has now been termed the dosage balance hypothesis [12–14].

The second difference between single-gene and genome duplication is the kind of adaptations each may give rise to. Interest in gene duplication is intense in evolutionary biology circles because, as Haldane recognized [15], duplication is a powerful means for generating genetic material with the potential for innovation. There are many models of duplicate gene evolution [16]: probably the most discussed are neofunctionalization [1, 2, 16], whereby one copy of a duplicate gene pair acquires a new beneficial function *after *the duplication, and subfunctionalization, where multifunctioned genes have their functions subdivided by duplication [17–19]. Since some of these subfunctions might themselves be novel and suffer from antagonistic pleiotropy (e.g., one subfunction cannot be optimized without detrimentally altering the other; [17, 20]) subfunctionalization can represent an important path to innovation. What genome duplication brings to this story is the potential for *multigene *novelties [21]: with a duplication of the entire genome to explore, evolution has more space to innovate. In this paper, we explore the evidence for multi-gene innovations in yeast and animals resulting from their respective WGDs [8, 22–25]. We then discuss in detail two key innovations that are associated with WGD: aerobic ethanol fermentation in yeast and increased complexity in the vertebrate developmental regulatory network. In so doing, we will remind ourselves of Francois Jacob's insight as to the mechanisms of evolution: the innovations produced are in keeping with the work of a tinkerer, not an engineer [26], and are contingent on their possessors' evolutionary history [27].

## 2. WGD and Single-Gene Innovations

The existence of neutral models of duplicate gene resolution [18, 19] and apparent examples of their action after WGD [28] means that, before pursuing multi-gene adaptations from WGD, it is worthwhile to pause and ask whether examples of single-gene innovations due to WGD are known. We do so even though those innovations may appear no different than what might be expected from an SSD event. As a matter of fact, there are good examples from yeast. For instance, consider the *S. cerevisiae *WGD-produced paralogs *GAL1* and *GAL3: *a sugar kinase and a regulator, respectively [29]. In the non-WGD *Kluyveromyces lactis,* the single ortholog of these two genes possesses both functions [30]. However, these two *ohnologs* [31] are not simply an example of neutral subfunctionalization: Hittinger and Carroll [20] have shown an adaptive conflict in the promoter of the *K. lactis* gene that was resolved by the gene duplication. In particular, it would be more “cost-effective” to have highly dynamic expression in the *K. lactis  GAL1  *gene, with strong repression in the absence of galactose. However, because this same locus also encodes the regulatory function performed by the Gal3 protein in *S. cerevisiae*, such strong repression would result in insufficient expression of* GAL1* to perform its regulatory function in the absence of galactose. Gene duplication allowed a decoupling of the expression levels of these two distinct functions. The WGD-produced duplication was thus exploited as the last step in the evolutionary development of a metabolic subsystem with a fine degree of transcriptional control.

## 3. Multigene Adaptations

**Figure 1:**
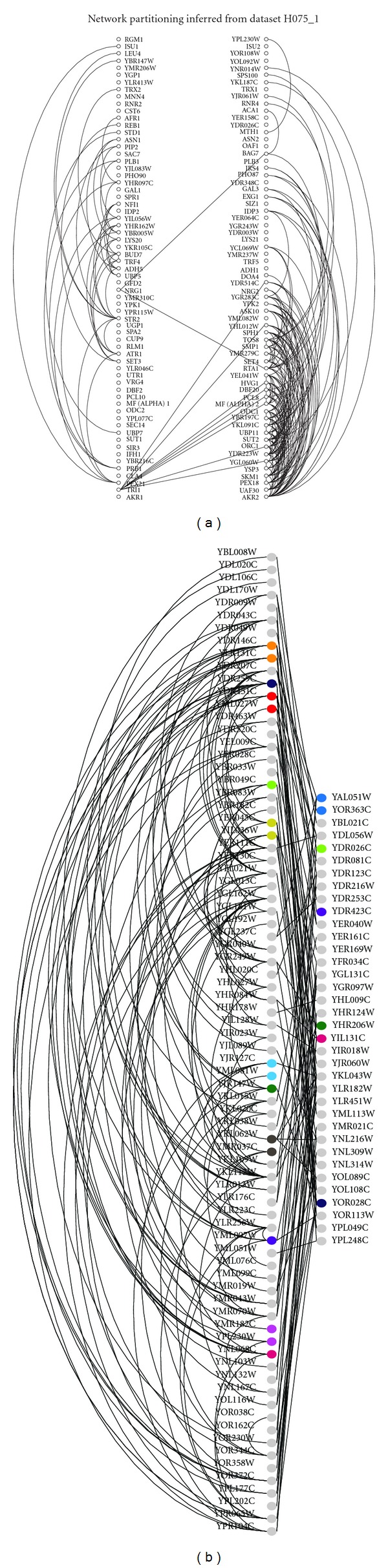
Network evolution after the yeast WGD. (a) The yeast coexpression networks show evidence of subfunctionalization after WGD. A co-expression network consisting of 65 pairs of WGD-produced paralogs (e.g., ohnologs) is illustrated. Each row contains a pair of ohnologs; edges join genes with co-expression correlation (Pearson's *r*) ≥0.75 across >200 microarray experiments. In each row, the position of the two ohnologs can be exchanged: we searched for the arrangement that minimized the number of interactions between the two columns (central diagonal edges). The number of such “crossing edges” is much smaller than what would be expected by chance (see [34]). (b) The above patterns are at least partly driven by changes in transcriptional regulation. We have previously shown that WGD-derived duplicated transcription factors have diverged considerably since WGD [45]. Here we show the relative lack of overlap between these duplicated regulators' functions. On the right are the transcription factors (TFs) that target other TFs but are not themselves targeted by a TF. On the left are the TFs that are regulated by other TFs. Duplicated TF pairs from WGD (e.g., ohnologs) are shown in the same color.

The most unique potential impact of genes duplicated at WGD, however, is not in single-gene adaptations. Instead, it is the potential for correlated changes across multiple genes resulting in altered cellular networks, including signal transduction and transcriptional regulatory networks. That such changes occur is indirectly suggested by the observations that duplicates from the yeast WGD are more likely to be part of protein complexes and more likely to share protein interaction partners than SSD duplicates [10, 32]. The products of such retained duplicates are also enriched for proteins regulated by phosphorylation [33]. Both observations are in keeping with the expectations of the dosage balance hypothesis [12]. Similarly, we have shown an example of coherent changes in the coexpression networks of *S.   cerevisiae*. To do so, we used an algorithm for detecting subdivided networks. This algorithm divides genes (connected by edges if they are coexpressed across multiple microarray experiments; [34]) into two columns, where each row consists of a pair of WGD-produced paralogs (Figure 1(a)). We then searched for the arrangement of genes that minimized the number of edges crossing between columns and compared that number to the number of such crossing edges seen in randomized networks. The relative paucity of crossing edges in the real network suggests *network* subfunctionalization, where groups of ohnologs are subdivided into two co-expression clusters [34].

### 3.1. WGD and the Crabtree Effect

While these global patterns of change after WGD suggest large-scale alterations, the best example of a change that can be at least provisionally tied to a phenotype is the evolution of the *Crabtree* effect. Baker's yeast is somewhat unusual in its metabolism: even when oxygen is available, it prefers to only partially oxidize glucose into ethanol rather than fully oxidize it into CO_2_ and water (the Crabtree effect; [35, 36]). This fermentative lifestyle is odd insomuch as it is energetically less favorable than the complete conversion of sugars into carbon dioxide (e.g., respiration). However, there is a general association between whether or not a yeast species possesses the ancient WGD and the Crabtree effect [37].

One clue to the source of this apparent paradox can be found in a group of duplicated genes from the WGD, all involved in the early stages of glucose metabolism. These genes include two glucose sensors (*SNF3* and *RGT2*), two glucose transporters (*HXT6/HXT1*), and two duplicate enzymes that catalyze the initial step of glycolysis (e.g., the hexokinases *HXK1* and HXK2). Strikingly, in all three ohnolog pairs, one member acts when glucose concentrations are low and the other when they are high [38–40]. A second piece of the puzzle is due to theoretical work on resource competition among organisms inhabiting a large but ephemeral environmental resource. Such competition among cells can actually favor lineages that rapidly oxidize glucose relative to their more efficient but slower-growing competitors [41–43]. This *tragedy  of  the  commons* [44] occurs because even though the efficient cells are able to convert more glucose into energy, they pay for this efficiency in reduced temporal growth rates, meaning that the fast, wasteful, cells can come to numerically dominate the resource patch.

**Figure 2:**
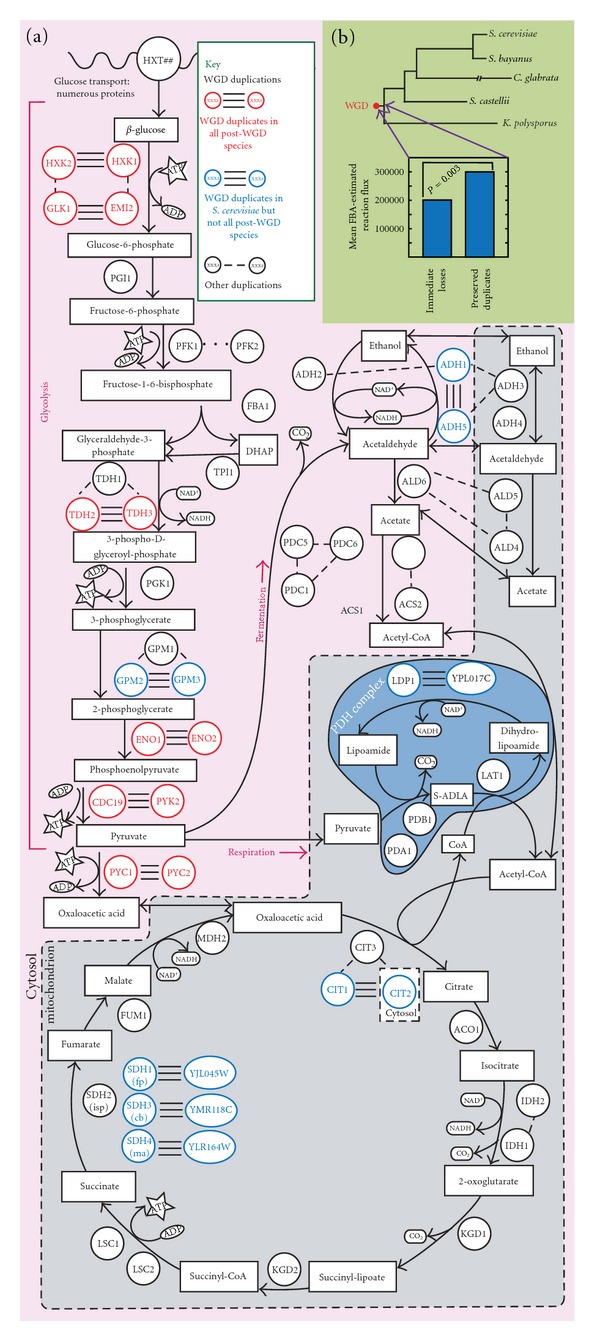
WGD and yeast carbon metabolism. (a) Illustrated are glycolysis, alcohol fermentation, and the mitochondrial TCA cycle. We denote the enzymes catalyzing a reaction with circled gene names. Products of SSD events are indicated by single lines joining the pair of enzymes. Enzymes duplicated at WGD are joined by three lines. WGD pairs in red are preserved in duplicate in four extant yeasts: *S.  cerevisiae*, *S.  bayanus*, *C.  glabrata*, and *S.   castellii*. Protein localization for *CIT*, *ADH,* and *ALD* is taken from Huh et al. [46]. We indicate the pyruvate dehydrogenase (PDH) multienzyme complex with a darker blue enclosure. There is a clear bias in where the duplicated enzymes lie, particularly if only those preserved in duplicate across four species are considered. From [47]. (b) Duplicated enzymes losses immediately after WGD were biased toward enzymes catalyzing low-flux reactions. The fluxes through all reactions in the yeast metabolic network [48] were computed under a variety of nutrient conditions as previously described [49]. Then, using our tool for estimating the timing of gene loss after WGD [50], we identified enzymes likely to have been lost along the short branch separating the WGD from the divergence of *K. polysporus* from the remaining four yeast species. We compared the fluxes of those enzymes to that of enzymes retained in duplicate along that same branch. The genes lost immediately after WGD were more likely to code for enzymes of low flux (*P* = 0.003, permutation analysis; unpublished data).

 Given these observations and expectations, we and others proposed that the yeast WGD had several effects on its patterns of glucose metabolism (Figure 2(a)). First, we proposed that the increase in gene copy number produced by the WGD gave rise (after some gene losses in other parts of the genome) to an increased flux through glycolysis [37, 47, 51, 52]. Second, because oxidative phosphorylation of pyruvate is constrained by oxygen concentrations and the spatial structure of the mitochondria, the WGD-possessing cells were required to redirect some of this increased glycolytic flux to the (previously anaerobic) fermentative pathways [47]. The result was likely to induce the sort of competitive situation between efficient and inefficient cells just described. A degree of independent confirmation to these ideas was provided by Van Hoek and Hogeweg [53], who were able to show computationally that similar WGD events modeled in modern *S.  cerevisiae* could also be expected to result in over retention of glycolytic enzymes and increased glycolytic flux. Recent work in our lab also supports this contention, showing that duplicate losses immediately after WGD were biased toward genes coding for low-flux enzymes (Figure 2(b), unpublished data).

If the WGD was in fact a trigger for moving *S.  cerevisiae* and its relatives down a path toward increasing Crabtree effect, we would expect it to have been followed by later evolutionary changes reinforcing this propensity. Indeed, at least two such post-WGD changes are known. First, in yeasts with the WGD, loss of *cis*-regulatory elements among the genes for the *mitochondrial* ribosomal proteins has de-coupled the expression of the cytosolic and mitochondrial ribosomal proteins [54]. This change had an important effect: *S. cerevisiae* can now upregulate production of cytosolic ribosomes independently of the mitochondrial ones, an outcome that increases the efficiency of aerobic fermentation by avoiding unnecessary ribosome synthesis in the quiescent mitochondria. The second example is a post-WGD *SSD* event in the alcohol dehydrogenase family. The result of this event was two specialized ADH loci, one for the synthesis of ethanol and a second isoenzyme responsible for the back-conversion of ethanol to pyruvate (once glucose is exhausted Crabtree yeasts can reimport and respire the ethanol they previously produced; [55]). Such specialization likely would only have been beneficial in the context of a preexisting WGD-produced Crabtree adaptation.

### 3.2. Other Examples of Coordinated Evolution in Post-WGD Yeasts

There are at least two other cellular subsystems in *S. cerevisiae* that show evidence of large-scale changes after WGD, although the details are less well understood than is the case for metabolism. First, in the transcriptional regulatory network, pairs of transcription factors duplicated at WGD, while still showing detectable similarities in their targets inherited from the WGD, have diverged considerably (Figure 1(b); [45, 56]). More interestingly, the cytosolic ribosomal proteins in *S. cerevisiae* were highly over-retained after-WGD [6], representing roughly 10% of all retained duplicates, despite being less than 4% of the pre-WGD genome [57, 58]. These duplicates are extremely curious in that many of them have undergone considerable gene conversion, such that, despite their divergence at the ancient WGD, they have virtually identical protein sequences in modern bakers' yeast [23, 58]. At first blush, this result could be explained in terms of selection for high copy number [59] and the dosage balance hypothesis. The story became mysterious, however, with the discovery that several of these paralogs, while nearly identical in protein sequence, have distinctly different knockout phenotypes [60–62]. In keeping with the idea of coordinated evolution among multiple paralogs, a number of these duplicated pairs show asymmetric specialization of one of the two ohnologs to expression in the developing bud of the yeast cell [61, 62]. We speculate that these ribosomal proteins will represent another example of a system-level specialization induced by the WGD. In this view, the rampant gene conversion is a result of the highly interactive nature of the ribosome. Thus, both paralogs must “fit” exactly into the complex ribosomal structure and what differs is not their protein function but their expression domain.

### 3.3. WGD and Evolutionary Innovations in Plants

WGD is rampant in plant genomes, particularly those of angiosperms [63, 64]. The systems and network biology of these events have recently been extensively reviewed [65–68], and we will not attempt to do justice to the subject here. However, we do note that while the complexity of plant biology makes identifying precise evolutionary trajectories quite difficult, there are several suggestive coincidences of timing between the origins of new traits and the duplication of regulatory genes involved in those traits [66]. For example, glucosinolates are a class of secondary metabolites, the diversity of which has become expanded in the model plant *Arabidopsis  thaliana* and its relatives. If one maps this expansion onto the phylogeny of these plants, it is curiously close to one of the *Arabidopsis* WGD events. Even more strikingly, several of the regulators and enzymes responsible for glucosinolate production in *Arabidopsis* have surviving duplications from that WGD [69]. More generally, we have recently shown [70] that the pattern of post-WGD duplicate retention in the *Arabidopsis* metabolic network seems to be driven by two different forces: a tendency to initially retain clusters of related enzymes (as would be expected under the dosage balance hypothesis) followed by a selective regime that appears to retain duplicates for reactions of high flux (similar to situation seen in *S. cerevisiae*).

### 3.4. 2R and the Remodeling of the Vertebrate Developmental and Signal Transduction Networks

Another example of WGD-induced functional innovation at the systems level concerns the vertebrate developmental toolkit and signal transduction engines. The metazoans, because they have bodies organized into distinct tissues, are clearly characterized by significant phenotypic complexity. They seem to have appeared about 640 million years ago and may have been preceded by other multicellular lineages of uncertain relationships [71]. On the basis of mitochondrial DNA sequence comparisons, the choanoflagellates have been identified as the closest single-celled animal relatives [72, 73] with the basal metazoan being either the placozoans [74–76] or the sponges [77, 78]. Although the role of WGD in metazoan evolution is not fully understood, several examples of WGDs among the vertebrates have been identified [21]. These include two rounds (2R) of genome duplication at the base of vertebrates (2R-WGD; [25]), the fish-specific genome duplication (FSGD; [4, 79, 80]), and WGDs in the genus *Xenopus* [81].

Despite their phenotypic complexity, animals' gene content is not vastly greater than that of other organisms [82, 83]. Part of the explanation for this relative paucity of extra genes is the nature of development, which occurs by sequential differentiation in bifurcating cell lineages rather than through entirely distinct differentiation programs for each tissue. Nonetheless, the transformation to multicellularity must have been accompanied by appearance of new genes coding for adhesion molecules, extracellular matrix proteins (such as collagen), and cell-to-cell communication. Indeed, considerable progress has been made in identifying the novel signaling pathways involved in control of development and body plan formation [84]. In keeping with the theme of relatively little genome expansion coupled to the appearance of the metazoans, only a small fraction of the genes in the genome contribute to the development of the body plan. However, these genes make up a developmental toolkit that is strongly conserved across the eumetazoans. Transcriptional factors of particular interest are homeobox genes (Hox, ParaHox, EHGbox, and NK-like); KLF, Osr and Sp1/Egr genes, tlx, Snail, and slug zinc-finger proteins; MASH, myoD, mef, hairy, and twist helix-loop-helix transcriptional factors; T-box transcriptional factors [85–87]. These transcriptional regulators interact with the outside world through signal transduction pathways, the most important of which are those employing transforming growth factor-*β* (TGF-*β*), Wnt, Notch, Hedgehog, Toll, tyrosine kinase receptors, the nuclear hormone receptors, and the G-protein-coupled receptors. The identification of the shared toolkit of signalling pathways underlying animal development is a key discovery of modern biology. Following this work, we have recently found that the vertebrate signal transduction engine was highly modified by the 2R-WGD [88], suggesting that some of the complexity of vertebrates may have required the innovative capacity of WGD [89].

### 3.5. Gene Duplications in the Transforming Growth Factor-*β* Pathway

Our initial study, focused on the TGF-*β* pathway, provided early evidence of the impact of the 2R-WGD on vertebrate signaling. This signaling pathway has been long recognized as one of the most fundamental and versatile in metazoans, with central roles in development, organogenesis, stem-cell control, immunity, and cancer [90]. After an investigation of 33 genomes, we showed that the evolution of the TGF-*β* pathway in animals can be best explained according to the 2R model, with additional duplications in teleost fishes [91]. The components of the core pathway (both receptors and Smads) expanded dramatically and permanently at the base of vertebrates as a result of the 2R-WGD. In particular, four ancestral Smads (an I-Smad, a Co-Smad, and two R-Smads of the BMP and TGF-**β* sensu  stricto* channels) gave rise to the eight known Smads of the human genome, classified as two TGF-*β* *sensu  stricto* (Smad2,3) and three bone-morphogenetic-protein- (BMP-) type (Smad1,5,8) receptor-activated Smads (R-Smads), one common mediator Smad (Co-Smad; Smad4), and two inhibitory Smads (I-Smads; Smad6,7).

### 3.6. General Expansion of Signaling Pathways after 2R

In a more general analysis, we found that the 2R-WGD affected the overwhelming majority (three quarters) of human signaling genes, with the strongest effect on developmental pathways involving receptor tyrosine kinases, Wnt and TGF-*β* ligands, GPCRs, and the apoptosis pathway. Unlike genes deriving from recent tandem duplications, genes retained after 2R were enriched in protein interaction domains and multifunctional signaling modules of Ras and MAP-kinase cascades. The set of human 2R-ohnologs (2ROs), corresponding to 9,958 unique Entrez Genes, is enriched in many classic signaling domains (such as tyrosine and serine/threonine kinase domains, the seven-transmembrane receptor domains of the rhodopsin and secretin families, and the Ras family domain), as well as well-known protein interaction domains, including the SH2, SH3, PTB, and PDZ domains.

 PDZ domains are particularly interesting as they are abundant in vertebrate neuronal synapses, serving as scaffolds for the assembly of large neurotransmission signaling complexes [92]. Thus, these results suggest that 2R may have provided evolutionary material for subsequent changes in vertebrate brain development. Further evidence for this contention came when we found that 2ROs are preferentially expressed in Gene Expression Atlas samples associated with brain and nervous tissue. These brain-expressed 2ROs are also enriched in Gene Ontology (GO) terms related to synaptic transmission. Studies in fly and mouse have shown that vertebrate synapses are more complex than those of invertebrates [93]: it is thus intriguing to speculate as to a role for 2R in inducing this phenomenon.

**Figure 3:**
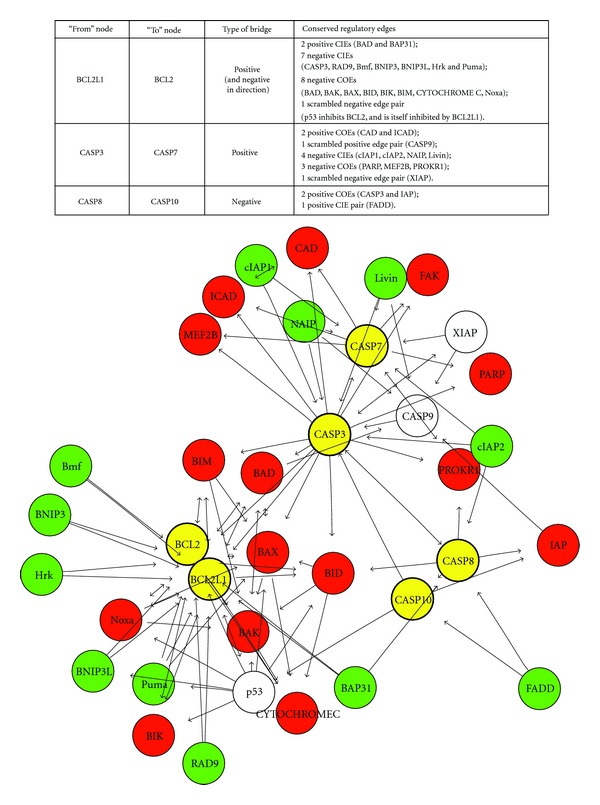
2R-ohnologs in the apoptosis pathway. A network diagram of the vertebrate apoptosis pathway is shown with pairs of 2R-ohnologs (2ROs) highlighted in yellow. There are three 2RO pairs in the subnetwork: BCL2L1 and BCL2; CASP3 and CASP7; CASP8 and CASP10. Nodes are color coded: yellow signifies nodes mapping to 2ROs, while green and red signify those mapping to CIEs and COEs, respectively (see text). CASP8 and CASP10 are initiator caspases. CASP3 and CASP7 are executioner-caspases functioning downstream of these initiator caspases. IAPs are apoptosis inhibitors. The balance between antiapoptotic BCL2 and BCL2L1, and proapoptotic BAD, BAK, BAX, BID, BIM, and Puma, and Noxa determines the final activity of the intrinsic pathway of apoptosis. From [88].

 Another potential source of vertebrate neuronal complexity is their use of apoptosis to shape brain structures and compartments. We found that the apoptosis pathway was dramatically remodeled through 2R [88].  Figure 3 illustrates the complex topology of the human apoptosis signaling subnetwork created by 2R [88]. It is clear that coordinated duplications of caspases resulted in a substantial evolutionary novelty. Moreover, the complexity of the evolutionary changes introduced by 2R is best appreciated by examining the conservation of regulatory interactions (directed edges in the network). To better illustrate changes in network topology induced by the 2R-WGD, we subdivided the conserved edges into those originating from a shared regulator and acting on a pair of 2ROs (conserved incoming edges—CIEs), and those originating from a paralogous pair directed towards a shared target (conserved outgoing edges—COEs). CIEs suggest a common conserved regulator, located upstream in terms of information flow. In contrast, COEs indicate evolutionary conservation of a common regulatory target, located downstream (Figure 3).

Finally, while many genes for ancient cellular functions were not retained in duplicate after 2R, the genes of the cell cycle are an exception to this rule (an interesting link to the overretention of cyclins after the yeast WGD; [8]). Most cyclins, including key cell cycle-regulating groups A, B, and D, underwent diversification at the base of vertebrates and are represented by between two and four vertebrate-specific paralogs derived from the 2R-WGD [88]. Similarly, cyclin-dependent kinases, cyclin-dependent kinase inhibitors, and orthologs of the *S.  pombe* WEE1 inactivator of the CDK/cyclin complex (Wee1 and Wee2) were also retained in duplicate after 2R [88]. Strikingly, cyclins D1-D3 respond to extracellular mitogens, cytokines, hormones, and juxtacrine ligands, providing an interface between signal transduction and the cell cycle. These cyclins then pair with CDKs 4 and 6, driving the transition to G1 [94–96]. It would be very informative to test if cyclins D1-D3 and CDK4/6 simply increase robustness of the cell cycle. If not, there may be functional differences between the 2R-derived cyclin D/CDK complexes in terms of the upstream signaling pathways they integrate or the downstream target genes they activate [88].

### 3.7. 2R and Vertebrate Complexity

In contrast to the predictions of the dosage balance hypothesis, vertebrate genes having developmental expression were more likely to revert to single copy after whole-genome duplication [97, 98]. However, this observation may be qualified by the fact that, after the FSGD, almost all retained duplicates have diverged in spatial and/or temporal expression during embryogenesis, and many were key developmental genes that function as transcription factors or signaling molecules during embryogenesis [99]. These general trends of retention and expression change, as well as the above functional analyses, clearly indicated that 2R fundamentally altered vertebrates' signaling pathways and cell cycles [88]. In consequence, it may have set the stage for the emergence of other key vertebrate evolutionary novelties (such as complex brains, the circulatory system, or heart, bone, cartilage, musculature, and adipose tissues; [71, 100]).

 It should also be noted that the methodology used in these studies of the 2R-WGD [88] precluded an investigation of the amphioxus genome, as this genome was not included in release 6 of the TreeFam database. However, in other studies, the genome of the cephalochordate *Branchiostoma  floridae *(e.g., amphioxus or lancelet) provided very strong evidence in support of the 2R hypothesis [101, 102]. Another strategically positioned pre-2R genome, that of sea urchin, is being developed as a developmental and systems biology model for understanding gene regulatory network evolution, which, together with the signal transduction pathways of this species, has been particularly well annotated [103–105]. Comparisons of sea urchin's developmental regulatory networks with those of vertebrates is likely to reveal further insights into the impact of the 2R-WGD.

## 4. Concluding Thoughts

The broader significance of these changes for our understanding of the forces and mechanisms driving the evolutionary process could well be extremely significant. Firstly, we propose that WGDs, like human technical innovations such as the railroad, greatly expand of genotypic and phenotypic space that might be explored by evolution. For example, the 2R quadrupling of components of the vertebrate signaling network not only immediately expanded the available space of signaling network states, but also kick-started rapid co-evolution of nodes into novel topologies during the subsequent “diploidization.” We have also recently proposed that WGD has an important role in evolutionary transitions by relaxing epistatic constraints [70], effectively increasing the size of the neutral genetic space in which innovation can occur [106]. Secondly, an exciting possibility exists that at least some WGDs may be instantaneous speciations: if so, they would be evolutionary events whose occurrence is somewhat in contrast to an exclusively gradualist view of evolution. Early authors of modern synthesis, coming from background in population genetics, were perhaps overly wedded to gradualism, where natural selection acts on small variations in large populations. The molecular mechanism of WGDs is most likely auto- or allopolyploidy. WGDs could therefore be interpreted as saltations, that is, sudden evolutionary changes occurring within a single generation. However, population genetic processes are of course of central importance during subsequent re-diploidization. During that gradual process of duplicate loss over millions of years, there may be losses driven by natural selection acting to fix null mutants for duplicated loci, a process which fits well with Neo-Darwinian views.

In a related vein, gene duplications may have a role in enhancing robustness—the organism's resilience to genetic or environmental perturbations [107]. At the simplest level, duplication provides short-term robustness through genetic “backups.” However, WGDs could also lead to an increase in distributed robustness, which is a consequence of the existence of multiple solutions to the same biological problem. A well-known example of this idea is the redundant paths through metabolic networks that confer robustness [48, 108]. It is fairly straightforward to envisage an analogous situation in signal transduction or the cell cycle: multiple regulatory mechanisms could in that case increase the level of control, allowing, for instance, the development of complex vertebrate embryos with many novel organs and tissue types.

 Genome duplication might have also facilitated innovation in other ways. For instance, the establishment of crosstalk between signaling pathways [109] may have resulted from WGD. The post-WGD redundancy would have allowed the partial subdivision of duplicated pathways, resulting in a network of a higher degree of connectivity and robustness. Thus, it is striking that few novel signaling genes emerged through post-2R events [88], since SSD events lack the opportunity for this type of change. Another area for future investigation is the impact of 2R-WGD on non-coding genes [110]. Published studies and our own observations indicate that no preferential retention of miRNA genes can be attributed to 2R-WGD [111]. Instead, functional innovation in miRNA regulation appears to have occurred during the more recent mammalian diversification [112]. This suggests a model where major evolutionary transitions exploit expansions in different classes of genomics elements: protein-coding genes at the transition to vertebrates and miRNA genes during diversification of mammals.

It is tempting to hypothesize that gene duplication can initially promote redundancy of system parts, allowing evolutionary tinkering, while genome duplications are correlated with an increase in distributed robustness. In the future, we propose testing this hypothesis by asking if more WGD-produced duplications are found in distinct signaling pathways when compared to SSD gene duplicates of similar age. More generally, both redundancy and robustness provide the evolutionary space for adaptations, and there are suggestions that WGD facilitated the colonization of novel environments and ecological niches [52, 113].

Genome duplication undoubtedly represents a tremendous evolutionary opportunity: the release of epistasis alone that results from WGD may have important implications [45]. However, as the examples described here suggest, the resulting innovations are unlikely to fit neatly into the neofunctionalization/subfunctionalization paradigm [16, 114], nor are they likely to be fully understood without a detailed knowledge of the cellular systems in which they are active.
